# Electric Ablation with Irreversible Electroporation (IRE) in Vital Hepatic Structures and Follow-up Investigation

**DOI:** 10.1038/srep16233

**Published:** 2015-11-09

**Authors:** Xinhua Chen, Zhigang Ren, Tongyin Zhu, Xiongxin Zhang, Zhiyi Peng, Haiyang Xie, Lin Zhou, Shengyong Yin, Junhui Sun, Shusen Zheng

**Affiliations:** 1 The Key Laboratory of Combined Multi-organ Transplantation, Ministry of Public Health; The Department of Hepatobiliary and Pancreatic Surgery, The First Affiliated Hospital, School of Medicine, Zhejiang University, Hangzhou 310003, China; 2 The Collaborative Innovation Center for Diagnosis and Treatment of Infectious Diseases, Zhejiang University, Hangzhou, Zhejiang, 310003, China

## Abstract

Irreversible electroporation (IRE) with microsecond-pulsed electric fields (μsPEFs) can effectively ablate hepatocellular carcinomas in animal models. This preclinical study evaluates the feasibility and safety of IRE on porcine livers. Altogether, 10 pigs were included. Computed tomography (CT) was used to guide two-needle electrodes that were inserted near the hilus hepatis and gall bladder. Animals were followed-up at 2 hours and at 2, 7 and 14 days post-treatment. During and after μsPEF ablation, electrocardiographs found no cardiovascular events, and contrast CT found no portal vein thrombosis. There was necrosis in the ablation zone. Mild cystic oedema around the gall bladder was found 2 hours post-treatment. Pathological studies showed extensive cell death. There was no large vessel damage, but there was mild endothelial damage in some small vessels. Follow-up liver function tests and routine blood tests showed immediate liver function damage and recovery from the damage, which correlated to the pathological changes. These results indicate that μsPEF ablation affects liver tissue and is less effective in vessels, which enable μsPEFs to ablate central tumour lesions close to the hilus hepatis and near large vessels and bile ducts, removing some of the limitations and contraindications of conventional thermal ablation.

Hepatocellular carcinoma (HCC) is the sixth-most-common malignancy worldwide, with half of the deaths from the disease occurring in China^1^. In China, hepatitis B virus (HBV) infection and fungal aflatoxin B1 exposure cause the high incidence of HCC. According to the Chinese cancer registry annual report, released in April 2014 by the National Central Cancer Registry (NCCR), HCC is still one of the most common cancers and leading causes of cancer deaths in China. HCC can be cured by liver transplantation (LT), liver resection (LR), or local ablation therapy^2^,^3^. More than 80% of HCC patients are diagnosed at advanced stages when the tumours cannot be surgically removed^4^. LT is regarded as the best therapeutic choice for patients who meet the Milan criteria^5^,^6^. However, patients who do not meet the Milan criteria are often dropped from waiting lists because of the shortage of donor organs. Our centre cautiously used the Hangzhou criteria to expand the candidate pool and increase the number of patients who could benefit from LT^7^,^8^, but prospective follow-up is ongoing to evaluate long-term HCC recurrence. Advanced cases of HCC need further pre-transplantation bridge therapy to downstage the tumour to meet LT criteria^9^,^10^, and the development of novel local ablation approaches for unresectable and non-transplantable HCC patients is urgently needed.

Various local ablation techniques have been used to ablate HCC^11^,^12^,^13^,^14^,^15^. Among them, radiofrequency ablation (RFA) is the most widely used method. RFA is easy, safe, cost-effective and minimally invasive^16^,^17^. However, RFA has major side effects, such as thermal damage to surrounding structures. When ablation is incomplete, residual tumours and satellite nodules will grow^17^,^18^,^19^. Lesions close to the gallbladder, stomach, bowel and heart can be difficult to treat with RFA. Therefore, new non-thermal ablation methods are greatly needed^2^,^20^.

In contrast to thermal ablation techniques, microsecond-pulsed electric fields (μsPEFs), also known as irreversible electroporation (IRE), are a novel non-thermal ablation technique^2^,^21^. This method releases electric field energy in a series of microsecond-duration pulses to ablate the tumour^21^,^22^. No significant heat is generated during the procedure^23^,^24^.

Previous studies have demonstrated that μsPEFs can effectively ablate HCC in mice by causing necrosis^2^,^25^. However, there are no preclinical trials with strict controls and systematic follow-up. This preclinical study was performed to observe the feasibility and safety of μsPEF ablation on porcine models and related vital organ functions by using a commercially available pulsor for a standard single procedure. The comprehensive follow-up study is particularly focused on (1) cell morphological characteristics; (2) histopathology and its correlation with ultra-structures and imaging findings at the same time points; and (3) liver function and haemanalysis, which show the systematic reaction to a locoregional treatment.

## Material and Methods

### Animal care

Ten female pigs (30 kg) were maintained by the Division of Experiment Animal Laboratory of Zhejiang University. All animals received appropriate humane care from certificated professional staff. Animal treatment protocols were approved by the Animal Care and Use Committee of Zhejiang University. The methods were carried out in accordance with the approved guidelines.

### Animal experiment setting

Animals were managed with general anaesthesia, mechanical ventilation, and a neuromuscular blockade to ensure complete paralysis. Two-needle electrodes were placed with computed tomography (CT) guidance. A radio-opaque probe tip was used for identification.

### The microsecond-pulsed electric field treatment

The animal treatment was performed with a pulse generator device (NanoKnife; AngioDynamics, Queensbury, NY). The μsPEF treatment parameters were 90 pulses of 3000 V and 20 A delivered during the absolute myocardial refractory period (after the R-wave on the electrocardiograph [ECG]) to prevent heart arrhythmias. The treatment plan was set according to the manufacturer’s instructions; 3000 V of electricity was delivered by a pair of monopolar probes for a single procedure.

### Computed tomography follow-up

Before treatment, a CT scan was performed to identify the ablation region. During treatment, CT was used to guide electrode placement. After treatment, plain CT without contrast medium was used to follow-up at 2 hours, 2 days, 7 days and 14 days. However, at 24 hours post-treatment, a contrast CT was also performed to examine portal vein thrombosis. Because the contrast medium may cause radiographic contrast nephropathy, which will affect the observation of kidney function, we performed the contrast CT only once.

### Biomedical follow-up

Before treatment, blood samples were collected to identify the baseline. After treatment, blood samples were collected serially to monitor liver function at 2 hours, 2 days, 7 days and 14 days.

### Pathology and ultra-structure follow-up

After treatment, the ablated liver tissue was dissected and fixed with formalin for haematoxylin and eosin (H&E) staining or 2.5% glutaraldehyde for transmission electron microscopy (TEM) in the Imaging Facility of Core Facilities, Zhejiang University School of Medicine, as noted in our previous description^26^,^27^.

## Results

### Animal safety

**Figure 1:**
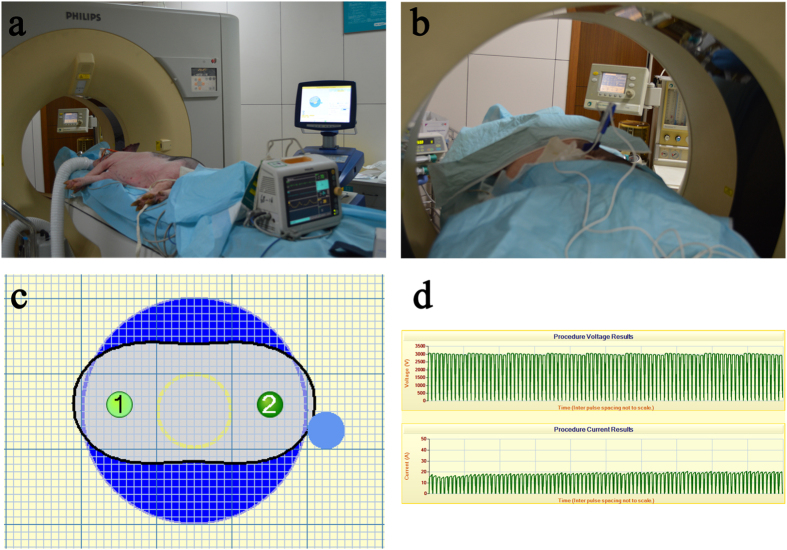
The microsecond-pulsed electric field treatment. (**a**) The animal treatment was performed in the interventional radiology suite using a pulse generator and an ECG monitor. (**b**) The two-needle electrodes were placed with CT scan guidance, and the treatment was performed under general anaesthesia. (**c**) The two-needle electrodes were placed at a distance predetermined by computer calculation and simulation to bracket the target ablation zone. (**d**) The electric ablation parameters were 3000 V, 20 A, 90 pulses, and 70 microsecond pulse duration.

A total of 10 pigs were included in the experiment (7 for treatment and 3 for control). The vital signs were stable during the treatment period in the interventional radiology suite, and the animals were monitored with an ECG and by an on-site anaesthetist (Fig. 1). Electrodes were placed near the right branch of the portal vein, the neck of gallbladder, and the hepatic artery. No heart arrhythmias or haemorrhage occurred during treatment.

### Complications

**Figure 2:**
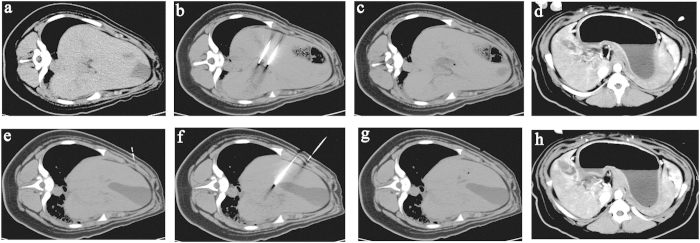
CT scan before and after treatment. The upper four CT scans are the liver before treatment (**a**), the two-needle electrodes inserted in the hilus hepatic (**b**), 2 hours after treatment (**c**) and 24 hours after treatment (**d**). The lower four CT scans are the gall bladder before treatment (**e**), the two-needle electrodes inserted near the neck of the gall bladder (**f**), 2 hours after treatment (**g**) and 24 hours after treatment (**h**). The CT images demonstrated a clear hypoattenuating area with a hyperattenuating rim in the ablated area.

As shown in Fig. 2, electrodes were inserted in the hilus hepatic (Fig. 2b) and near the neck of the gall bladder (Fig. 2f). CT images demonstrated a clear hypoattenuating area with a sharp hyperattenuating rim in the ablated area 2 hours after treatment (Fig. 2g) and 24 hours after treatment (Fig. 2h). These results confirmed those of the ECG monitor, which detected no cardiovascular events (e.g., supraventricular tachycardia, atrial fibrillation, and pneumothorax) during or after ablation. The CT scan detected no immediate complications. After 24 hours, a contrast CT did not find any portal vein thrombosis (Fig. 2).

### CT follow-up

Electrodes were placed near the hilus hepatis and vital hepatic structures, such as the right branch of the portal vein, the neck of gallbladder and the hepatic artery. After treatment, necrosis was found in the ablation area. There was no obvious evidence of aneurysm or thrombus formation. Figure 2 shows a sharp zone of necrosis in the centre of the ablation area and mild cystic oedema in the gall bladder 2 hours post- treatment. The connective matrix of the surrounding blood vessels remained intact.

### Pathological follow-up

**Figure 3:**
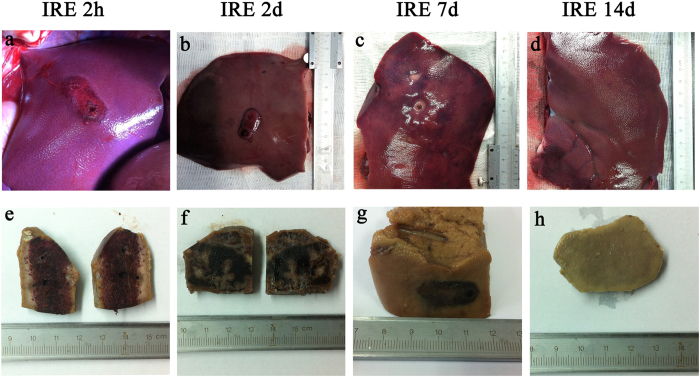
Gross pathologic sectioned specimen of the ablated porcine liver. Parts (**a**), (**b**), (**c**) and (**d**) show the discoloration caused by ablation on the dissected liver 2 hours, 2 days, 7 days and 14 days post-treatment, respectively. Parts (**e**), (**f**), (**g**) and (**h**) show the sharp demarcation of the ablated zone on the formalin-fixed liver. Intact vessels and bile ducts are seen in the area of ablation. The ablated zone showed vascular congestion and haemorrhagic change, with grossly intact hepatic morphology, 2 hours, 48 hours and 7 days after treatment. By day 14, the areas of vascular congestion and haemorrhage had resolved in the ablated zone. A well-demarcated margin was visualized between the ablated and non-ablated zones. The traversing vessels and bile ducts appeared intact within the ablated zones.

**Figure 4:**
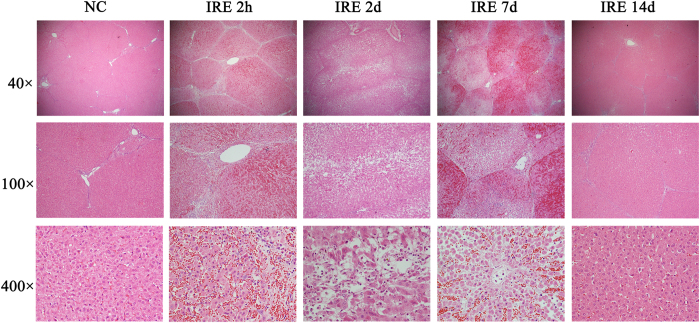
Haematoxylin and eosin (H&E) stained sections. Figure 4 shows haemorrhagic change with relatively intact hepatic morphology. Each hepatic lobule was visible and intact throughout the ablation area. The vessels and bile ducts appeared to have mild oedema without apparent structural destruction. On the 2-hour, 2-day and 7-day H&E slides, there was acute, extensive, and severe cell death. No viable cells were detected within the ablated area. However, the normal hepatic architecture was preserved. The ablation creates three layers: in the centre layer, where the electrode was inserted, there is haemorrhaging and severe necrosis; in the middle layer, there is coagulative type cell death; and in the outer layer, there are clusters of cell death. From 2 days to 7 days, the ablation area was filled with congestion from neutrophil and eosinophil infiltration. The larger vessels and bile ducts in the ablated area appeared structurally preserved. However, there was mild vaculitis, multifocal loss of endothelial integrity, oedema, separation of the tunica muscularis layers, and neutrophilic infiltration. The bile ducts showed signs of acute choledochitis with peridochal oedema. After 14 days, there was extensive hepatocellular regeneration in the ablation area.

**Figure 5:**
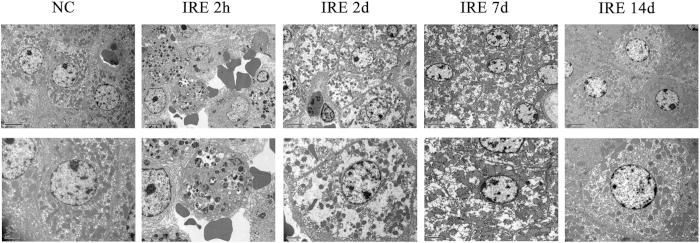
Transmission Electron Microscopy. Transmission electron microscopy (TEM) images with 1250 × magnification (upper) and 2500 × (lower). The control group showed integrity of the cell membrane and nuclear envelope. The cells exhibited a totally disorganized structure 2 hours after treatment and showed destruction 2 days after treatment. Necrosis was indicated 7 days after treatment. The cells recovered from the ablation and repopulated 14 days after treatment. In accordance with H&E, TEM showed areas of extensive and severe cell death, which was evidenced by a pyknotic and hyperchromatic nucleus and eosinophilic cytoplasm with vascular congestion and neutrophil infiltration 2 hours and 2 days after treatment.

Figure 3 shows the gross anatomy of the dissected tissue with the distinguishing feature of sharp ablation zones. Figure 4 shows the pathology of the ablated liver, marked by extensive cell death and with no microscopically viable cells in the ablation zone 2 hours to 2 days post-treatment. Endothelial damage in the blood vessels was found immediately after treatment. The endothelium of the vessels was affected, but not as seriously as the liver tissues in the ablation area. Re- endothelialization occurred 2 days post-treatment. After 14 days, the areas of vascular congestion and haemorrhage had resolved in the ablated zone. A well-demarcated margin was visualized between the ablated and non-ablated zones. The traversing vessels and bile ducts appeared intact within the ablated zones. The smooth muscle cells had repopulated 14 days after μsPEF ablation. In accordance with the gross anatomy and pathology, TEM (Fig. 5) showed an ablated liver characterized by extensive and severe cell death with a pyknotic and hyperchromatic nucleus and eosinophilic cytoplasm, vascular congestion, and neutrophil infiltration.

### Vital organ function follow-up

**Figure 6:**
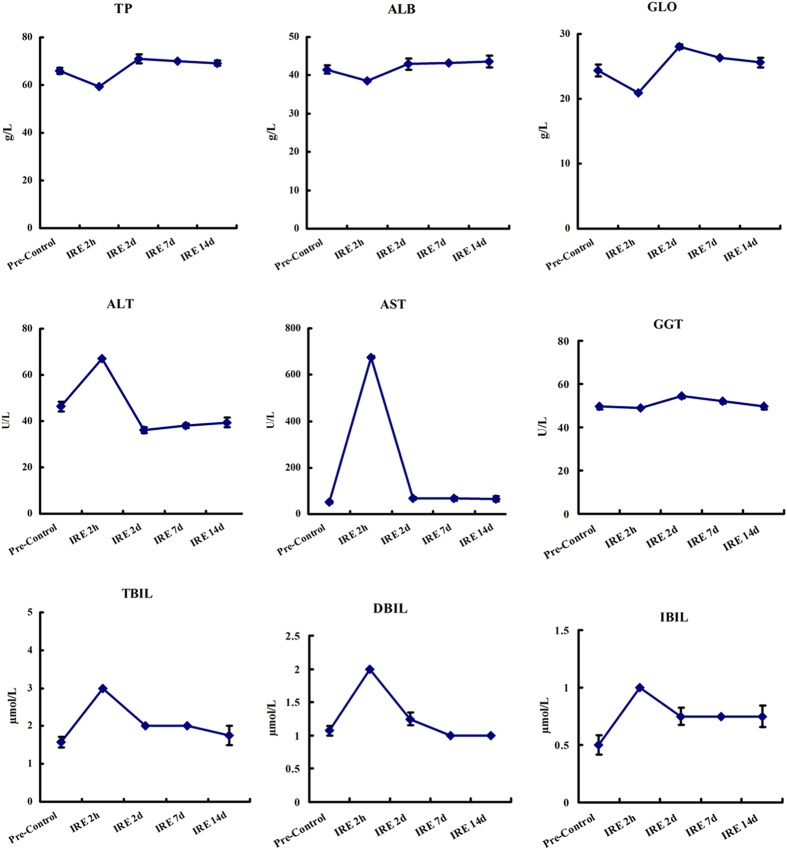
Liver function follow-up. Liver function was determined by testing total protein (TP), albumin (ALB), globulin (GLO), alanine aminotransferase (ALT), aspartate aminotransferase (AST), glutamyl transferase (GGT), total bilirubin (TBIL), direct bilirubin (DBIL), and indirect bilirubin (IBIL). The blood samples were collected at pretreatment and at 2 hours, 2 days, 7 days and 14 days post-treatment.

**Figure 7:**
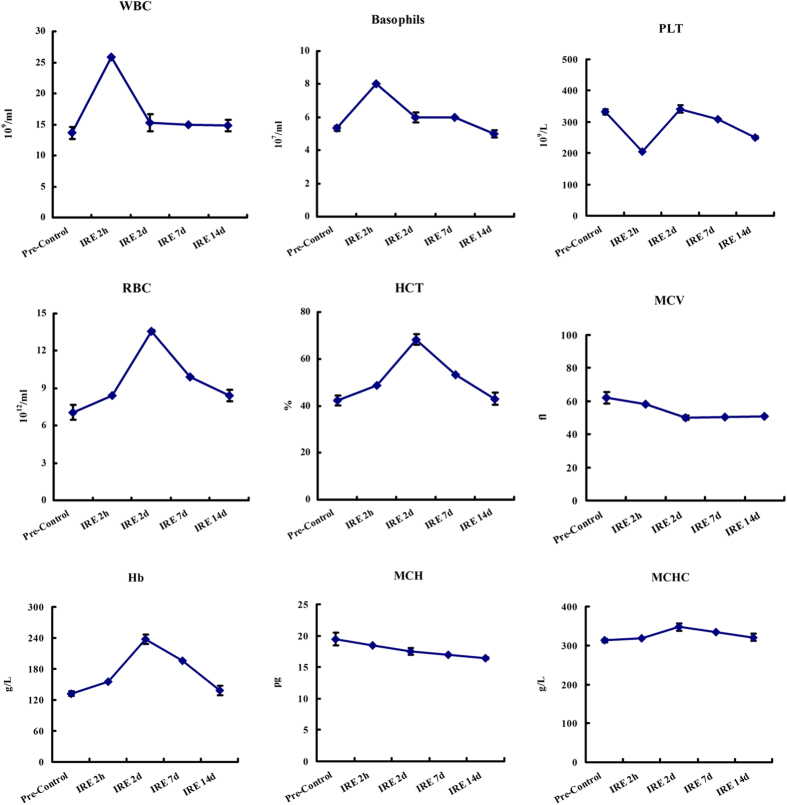
Blood count follow-up. The blood count was determined by testing white blood cells (WBC), basophils, red blood cell count (RBC), haemoglobin (Hb), haematocrit (HCT), mean corpuscular volume (MCV), mean corpuscular haemoglobin (MCH), and mean corpuscular haemoglobin concentration (MCHC). The blood samples were collected at pretreatment and at 2 hours, 2 days, 7 days and 14 days post-treatment.

Figure 6 shows the liver function follow-up, which was conducted by testing total protein, albumin, globulin, alanine aminotransferase, aspartate aminotransferase, glutamyl transferase, total bilirubin, direct bilirubin, and indirect bilirubin. Figure 7 shows the blood count follow-up, which was conducted by testing white blood cells, basophils, red blood cell count, haemoglobin, haematocrit, mean corpuscular volume, mean corpuscular haemoglobin and mean corpuscular haemoglobin concentration. These follow-up laboratory tests found that liver function damage occurred 2 hours to 2 days post-treatment. The enzyme and bilirubin counts rose from the baseline. In the meantime, the routine blood tests showed a mild inflammatory response. After 2 days, the increased enzyme and white blood cell counts began to drop. After 14 days, the abnormal results had returned to baseline.

## Discussion

IRE is a new ablation modality that can be applied in interventional radiology^16^. It delivers electric pulses to attain non-thermal focal ablation that spares vasculature and bile ducts and decreases the temperature-associated morbidity of conventional therapies^23^,^28^. Areas that were previously contraindicated for thermal techniques can now be ablated^28^,^29^,^30^,^31^,^32^.

Previous studies have indicated that IRE has the advantage of sparing vital structures, such as ducts^33^, nerves^34^ and blood vessels^35^ near the ablation zone. IRE was proposed decades ago^32^ and has been tested in several pre-clinical^36^,^37^,^38^,^39^ and clinical trials^28^,^40^,^41^ involving the pancreas^42^, kidneys^43^, liver^44^, brain^22^,^37^, and prostate^45^. The liver is the best organ for ablation. IRE has been shown to treat hepatic tumours in rats^46^ and rabbits^47^. It has also been shown to preserve hepatic veins and adjacent tissues in porcine livers^48^. Cardiac arrhythmias during IRE can be reduced by using electrocardiograph synchronization^49^, but they still need to be closely monitored^40^,^50^,^51^,^52^,^53^. IRE causes necrosis in the ablation area^33^,^54^ but preserves surrounding blood vessels and bile ducts^33^,^55^. Endothelial damage to blood vessels has been reported^54^, but re-endothelialization occurred within 7 days^56^.

The ablation effect of IRE on tumour cell death is well established, but the mechanism is still controversial. Both apoptosis^57^,^58^ and necrosis^23^,^46^ have been reported in previous studies. Apoptosis is defined as programmed cell death (PCD), which causes cells to shrink without inflammation and is characterized by bubble-like blebs on the cell membrane. In contrast, necrotic cells break with interior structures distending with inflammation and oedema. In the current study, under preset parameters and procedures from three different levels of pathological study, gross anatomy, H&E staining and TEM, we observed complete necrosis. Both apoptosis and necrosis can be seen as part of a spectrum of shared biochemical events that result in some form of cellular death. The IRE ablation causes destruction. According to our experience, the lesion conductivity and tumour volume are two crucial factors that decide the outcome of IRE treatment. A well-designed electrode and its precise placement are two preconditions needed to minimize damage to the surrounding healthy tissue.

This research systemically followed-up the ablation outcomes to provide more physiological data during the post-ablation period. There are several advantages of μsPEF ablation. (1) The liver tissue near the hepatic veins and/or portal pedicles can be accurately ablated. The vascular and biliary structures adjacent to the ablation area are not injured. The μsPEF releases short pulses over microsecond durations without heat accumulation and, thus, avoids thermal injury to the surrounding structures. It also preserves connective tissue and large blood vessels in the ablation area. (2) There were no complications after treatment, such as blood vessel thrombosis, bile duct injury, intestinal perforation, haemorrhage, haematoma, hepatic abscess, pneumothorax or arrhythmia. (3) Unlike thermal ablation techniques^14^, which destroy all normal and pathologic tissue and blood vessels in the ablation zone, μsPEFs ablate liver tissue in the ablation region while preserving large vessels and bile ducts. Pathological studies have shown that inflammation occurred and small vessels were affected. The damage recovered 14 days after treatment, supporting previous reports that IRE causes mild endothelial damage to blood vessels immediately after treatment^54^ but later produces signs of re-endothelialization^56^. (4) Our data shows that μsPEF ablation affects liver tissue and is less effective in the vessels comprising collagenous and elastic fibres, which enables μsPEFs to ablate central tumour lesions close to the hilus hepatis, larger blood vessels and bile ducts, removing some of the limitations and contraindications of conventional thermal ablation. The liver enzyme and white blood cell count can be used to determine the liver function damage and inflammation caused by ablation.

The commercially available instrument from the Angiodynamic Company delivers microsecond-duration pulses. Further study is needed to investigate the ablation mechanism. Our pathological and TEM results indicate that ablation causes cell death by necrosis instead of apoptosis. The results also show that μsPEFs can preserve large vessels and cause only mild damage to small vessels in the ablation zone, which can recover after two weeks. These characteristics are organ-dependent and affect IRE treatment outcomes. The electric properties of different tissue types, especially conductivity, may be the key factors that determine different influences^59^. The ablation effect of IRE depends on electric field strength and tissue properties^60^,^61^. The mechanism is still not clear and needs further investigation.

As visualization of the needles was the main obstacle we encountered, a CT scan offers advantages in the guidance of percutaneous IRE for HCCs not larger than 4 cm. To be fully covered by the two-needle electrode-produced electric field, we suggest that the ablation area meet the maximum ablation size criteria of less than 4 cm.

In conclusion, μsPEF ablation affects liver tissue and is less effective in vessels, which enables μsPEFs to ablate central tumour lesions close to the hilus hepatis and near large vessels and bile ducts, removing some of the limitations and contraindications of conventional thermal ablation.

## Additional Information

**How to cite this article**: Chen, X. *et al.* Electric Ablation with Irreversible Electroporation (IRE) in Vital Hepatic Structures and Follow-up Investigation. *Sci. Rep.* **5**, 16233; doi: 10.1038/srep16233 (2015).
